# Proangiogenic neutrophilic-myeloid-derived suppressor cells emerge via two parallel pathways in renal cell carcinoma and melanoma

**DOI:** 10.1186/2051-1426-1-S1-P159

**Published:** 2013-11-07

**Authors:** Jennifer Ko, Patricia Rayman, Dana Obery, Daniel Lindner, Ernest Borden, James Finke

**Affiliations:** 1 Cleveland Clinic, Cleveland, OH, USA

## 

Targeted therapy including anti-angiogenic therapy, kinase inhibitors, and immunotherapy dominate cancer drug development in renal cell carcinoma (RCC) and malignant melanoma (MM), where traditional methodologies have failed. Myeloid-derived suppressor cells (MDSC) may represent a mode of resistance to such treatments and require investigation in human patients. Here we explore the relationship between conventional neutrophils and neutrophilic-MDSC (nMDSC) in RCC and MM. We show that T-cell suppressive nMDSC consist of hypodense CD11b+CD15+CD14- immature CD16- myelocytes/metamyelocytes and mature CD16+ neutrophils co-precipitating with peripheral blood mononuclear cells (PBMCs) in patient blood (n=22) and tumors (n=16). Such cells were not significantly present in healthy blood donors (n=5). In parallel, conventional neutrophils co-precipitating with red blood cells (RBCs) were found to be uniquely immunosuppressive when isolated from RCC or MM patients (n=8), but not when isolated from healthy blood donors (n=5)(p<0.05). Both hypodense n-MDSC as well as conventional neutrophils from cancer patients expressed increased levels of pro-angiogenic molecules (n=4) and enhanced tumor-associated angiogenesis of RCC and MM xenografts in nude mice (p<.0001). This functional shift in mature neutrophils was modeled in vitro upon exposure of healthy donor derived neutrophils to tumor cell conditioned medium; and, was associated with an increase in neutrophil production of immunosuppressive arginase and reactive oxygen species, as well as an increase in proangiogenic molecule expression and in vivo angiogenic activity (n=3, p<.0001). The data support a role for nMDSC that are both T-cell suppressive and pro-angiogenic in RCC and MM cancer patients. Furthermore, our experiments suggest that n-MDSC arise via the parallel premature egress of bone marrow myeloid precursors as well as a functional switch in conventional neutrophil activity in cancer patients. Such activity is at least in part mediated via soluble tumor-derived factors which may circulate in patient blood. Ancillary strategies targeting myeloid cells may be an important part of future clinical trials to enhance the potency of targeted therapy.

